# Effects of Intermittent Hypoxia-Hyperoxia Exposure Prior to Aerobic Cycling Exercise on Physical and Cognitive Performance in Geriatric Patients—A Randomized Controlled Trial

**DOI:** 10.3389/fphys.2022.899096

**Published:** 2022-05-26

**Authors:** Tom Behrendt, Robert Bielitzki, Martin Behrens, Oleg S. Glazachev, Lutz Schega

**Affiliations:** ^1^ Department for Sport Science, Chair for Health and Physical Activity, Otto-von-Guericke University Magdeburg, Magdeburg, Germany; ^2^ Department of Orthopedics, Rostock University Medical Center, Rostock, Germany; ^3^ Department Human Physiology, Institute of Clinical Medicine, I.M. Sechenov First Moscow State Medical University, Moscow, Russia

**Keywords:** hypoxic conditioning, cognitive impaiment, dementia, aging, elderly

## Abstract

**Background:** It was recently shown that intermittent hypoxic-hyperoxic exposure (IHHE) applied prior to a multimodal training program promoted additional improvements in cognitive and physical performance in geriatric patients compared to physical training only. However, there is a gap in the literature to which extent the addition of IHHE can enhance the effects of an aerobic training. Therefore, the aim of this study was to investigate the efficacy of IHHE applied prior to aerobic cycling exercise on cognitive and physical performance in geriatric patients.

**Methods:** In a randomized, two-armed, controlled, and single-blinded trial, 25 geriatric patients (77–94 years) were assigned to two groups: intervention group (IG) and sham control group (CG). Both groups completed 6 weeks of aerobic training using a motorized cycle ergometer, three times a week for 20 min per day. The IG was additionally exposed to intermittent hypoxic and hyperoxic periods for 30 min prior to exercise. The CG followed the similar procedure breathing sham hypoxia and hyperoxia (i.e., normoxia). Within 1 week before and after the interventions, cognitive performance was assessed with the Dementia-Detection Test (DemTect) and the Clock Drawing Test (CDT), while physical performance was measured using the Timed “Up and Go” Test (TUG) and the Short-Physical-Performance-Battery (SPPB).

**Results:** No interaction effect was found with respect to the DemTect (*η*_*p*_^*2*^ = 0.02). An interaction effect with medium effect size (*η*_*p*_^*2*^ = 0.08) was found for CDT performance with a higher change over time for IG (*d* = 0.57) compared to CG (*d* = 0.05). The ANCOVA with baseline-adjustment indicated between-group differences with a large and medium effect size at post-test for the TUG (*η*_*p*_^*2*^ = 0.29) and SPPB (*η*_*p*_^*2*^ = 0.06) performance, respectively, in favour of the IG. Within-group post-hoc analysis showed that the TUG performance was worsened in the CG (*d* = 0.65) and remained unchanged in the IG (*d* = 0.19). Furthermore, SPPB performance was increased (*d* = 0.58) in IG, but no relevant change over time was found for CG (*d* = 0.00).

**Conclusion:** The current study suggests that an additional IHHE prior to aerobic cycling exercise seems to be more effective to increase global cognitive functions as well as physical performance and to preserve functional mobility in geriatric patients in comparison to aerobic exercise alone after a 6-week intervention period.

## 1 Introduction

Even in the absence of chronic disease, aging is accompanied by a variety of considerable biological changes that can negatively affect the structure and function of the organic systems (e.g., brain and skeletal muscle) and in turn the individual’s capabilities (e.g., cognitive and physical performance) (Fjell and Walhovd, 2010; Seidler et al., 2010; Trombetti et al., 2016). In this regard, it has been shown that normal aging as well as various clinical disease (e.g., dementia) are associated with alterations in brain structure (Discoll et al., 2009; Kalpouzos et al., 2012) and the deterioration of cognitive performance, such as global and specific cognitive functioning (e.g., processing speed, visuospatial ability, and executive function) (Hoogendam et al., 2014; O’Shea et al., 2016). Furthermore, there is solid evidence that physical capacity (e.g., muscle strength and mass (Manini and Clark, 2012; Keller and Engelhardt, 2013), peak or maximum oxygen uptake (Letnes et al., 2020; Fuller et al., 2021)) decreases as a function of age. Epidemiological studies have found that the age-related loss of muscle strength (dynapenia) (Sillanpää et al., 2014; Gouveia et al., 2020) and peak oxygen uptake (Fleg et al., 2005; Arnett et al., 2008) were closely related to the decline in physical performance (e.g., mobility and balance). This was especially evident in people aged over 70 making it difficult for the elderly to engage in activities of daily living (Shephard, 2009; Hairi et al., 2010). Remarkable, there is evidence for a relationship between cognitive and physical performance in older adults with and without cognitive impairment or disease (McGough et al., 2011; Bruce-Keller et al., 2012; Ikegami et al., 2019). Results of longitudinal studies assessing the temporal relationship between cognitive and physical performance changes showed, however, inconsistent results demonstrating that 1) higher levels of cognitive performance predicted slower declines in physical performance (Atkinson et al., 2010; Taekema et al., 2012; Gothe et al., 2014), or 2) vice versa (Inzitari et al., 2007; Best et al., 2016), and 3) cognitive and physical performance worsened together with age rather than exhibiting an unidirectional relationship (Gale et al., 2014; Krall et al., 2014; Ikegami et al., 2019). However, it has been reported that measures of cognitive and physical performance are predictors for all-cause mortality, disability, and the onset and progression of dementia and could therefore be used as a surrogate endpoint to quantify the efficiency and health benefits of specific treatments in older adults (Wilkins et al., 2013; Legrand et al., 2014; Takata et al., 2014; Pavasini et al., 2016; Hayat et al., 2018; Doi et al., 2019).

Hence, research frequently aims to discover efficient interventional treatment strategies that delay age-related changes and ensure the maintenance of cognitive and physical performance. In general, physical training (also referred to as chronic physical exercise, i.e., when physical exercise is conducted regularly in a planned, structured, and purposive manner with the objective to increase or maintain individual capabilities in one or multiple fitness dimensions (Herold et al., 2019)), including aerobic, resistance, flexibility, and balance training, has been recognized as a valuable intervention strategy to counteract age-related changes (Singh, 2002; Izquierdo et al., 2021).

In the past, new methods have been studied regarding their potential to optimize the physiological adaptations and benefits of physical training by manipulating the environmental conditions (e.g., continuous aerobic training under normobaric hypoxia) (Pramsohler et al., 2017; Przyklenk et al., 2020). In this context, intermittent hypoxic exposure has gained considerable popularity as an effective non-pharmacological approach to increase the efficiency of physical training and thus to treat various age-related changes in humans (Verges et al., 2015; Millet et al., 2016; Serebrovskaya and Xi, 2016; Yeo, 2019). Intermittent hypoxic exposure represents a non-invasive method, which is characterized by repeated resting exposures to brief periods of (normobaric) hypoxia interspersed with normoxic periods. From a physiological point of view, periodic and alternating exposure to hypoxia and normoxia triggers the stabilization of hypoxia inducible factors (Semenza, 2009) and the upregulation of reactive oxygen species (Clanton, 2007). In particular, these factors are known to induce the expression of proteins (e.g., erythropoietin, nuclear factor erythroid 2-related factor 2 [Nrf2], vascular endothelial growth factor, nitric oxide synthase, peroxisome proliferator-activated receptor gamma coactivator-1α, and glycolytic enzymes) responsible for various adaptations that are implicated in the deceleration and prevention of age-related changes and chronic diseases, respectively (Mallet et al., 2020; Sies and Jones, 2020; Burtscher et al., 2021). Specifically, animal and human studies have shown that the synthesis of these proteins suppressed inflammation (Sifringer et al., 2009), activated angiogenesis (Hickey and Simon, 2006; Wahl et al., 2013), improved vascular endothelial function (Vedam et al., 2009; Muangritdech et al., 2020), stimulated mitochondrial biogenesis (O’Hagan et al., 2009; Scarpulla, 2011), and maintained glucose homeostasis (Choi et al., 2005). Therefore, it was hypothesized that the adaptive responses to chronic intermittent hypoxic exposure could elicit neuroprotection (Manukhina et al., 2016; Iyalomhe et al., 2017; Mallet et al., 2020; Burtscher et al., 2021) and mitigate the loss of muscle mass as well as strength (Millet et al., 2016; Jung et al., 2021). These adaptations can increase or maintain cognitive and physical performance during the aging process. In accordance with this hypothesis, Wang et al. (2020) reported in a pilot study that global cognitive function and short-term memory (operationalized by Mini-Mental State Examination [MMSE] and digital span test, respectively) were increased after 8 weeks of intermittent hypoxic exposure in older patients (58–78 years) with amnestic mild cognitive impairment. Furthermore, in another pilot study conducting an intervention period of 6 weeks, Schega et al. (2013) found that additional intermittent hypoxic exposure prior to a strength training is more effective for improving information processing speed and attention (operationalized by d2-test) compared to strength training alone in healthy, physically active older people aged between 60 and 70 years. There is also evidence that 3 weeks of regular exposure to intermittent hypoxia alone improved exercise tolerance and peak oxygen uptake in recreational active middle aged to old people with and without coronary artery disease (50–70 years) (Burtscher et al., 2004) and chronic obstructive pulmonary disease (30–75 years) (Burtscher et al., 2009). In the last decade, a modified hypoxia method that combines hypoxic and hyperoxic periods, namely intermittent hypoxic-hyperoxic exposure (IHHE), has emerged. Supported by animal studies, it has been hypothesized that replacing normoxia by hyperoxia can increase the adaptive responses to the intermittent hypoxic stimulus through upregulation of reactive oxygen species (Gonchar and Mankovska, 2012; Sazontova et al., 2012) and more rapid protective effects in the membrane structure of the heart, brain, and liver against free radical oxidations (Arkhipenko et al., 2005). Studies have further found that the mitochondria respiratory chain is one of the main intracellular sources of reactive oxygen species (e.g., hydrogen peroxide [H_2_O_2_]) in human cells, which are generated in response to the incomplete electron reduction of molecular oxygen (Murphy, 2009; Bae et al., 2011). Reactive oxygen species disrupt the link between Nrf2 and its repressor Kelch-like ECH-associated protein 1, thus releasing Nrf2 to enter the nucleus, where it triggers intracellular redox signalling cascades, which activate, among others, antioxidant and anti-inflammatory genes (Sazontova et al., 2012; Burtscher et al., 2021; Mallet et al., 2021). Although the exact molecular mechanisms triggered by IHHE are not fully understood, reactive oxygen species-induced signalling pathways are thought to be enhanced when hypoxia and hyperoxia alternate in place of normoxia (Sazontova et al., 2012; Burtscher et al., 2021). Thus, the intensity of signals that trigger adaptations might be increased by replacing normoxic by hyperoxic periods without affecting the hypoxic component. Recently, placebo-controlled trials examined the effect of IHHE on cognitive (Bayer et al., 2017a; Serebrovska Z et al., 2019) and physical performance in humans (Bayer et al., 2017a; Glazachev et al., 2017; Susta et al., 2017; Dudnik et al., 2018; Glazachev et al., 2019). For instance, Bayer et al. (2017a) investigated the effect of IHHE (i.e., alternating exposures to 4–7 min of 10%–14% of oxygen and 2–4 min of 30%–40% of oxygen for 4–8 cycles per session) in addition to an individualized multimodal training program (consisting of 30 min of physiotherapy procedures, 60 min of occupational therapy, and 20 min of aerobic cycling training) on cognitive and physical performance in older geriatric patients (64–92 years). At the end of the 5–6 week intervention, patients who had conducted IHHE three times a week in addition to a multimodal training program showed greater improvements in global cognitive function (i.e., Dementia Detection Test [DemTect] and Clock Drawing Test [CDT]) as well as physical performance (i.e., 6-min walk test) than those who had performed the same multimodal training program combined with sham IHHE (i.e., breathing normoxic air) (Bayer et al., 2017a). Thus, previous studies suggest that intermittent hypoxic or hypoxic-hyperoxic exposure can be used to increase the adaptive response of a strength training (Schega et al., 2013) or a multimodal training program (Bayer et al., 2017a) in older people, respectively. However, so far and to the best of our knowledge, no study has examined the chronic effect of IHHE added prior to aerobic exercise on cognitive and physical performance in older adults.

The positive effect of aerobic training alone on brain health has been reported by several meta-analytic investigations indicating that aerobic training improves cognitive performance, including global and specific cognitive functioning in non-demented older adults (Smith et al., 2010; Barha et al., 2017; Bouaziz et al., 2017; Northey et al., 2018) as well as in patients with mild cognitive impairment (Zheng et al., 2016) and dementia (Groot et al., 2016). Moreover, it is well established that aerobic exercise elicits large improvements in physical capacity, estimated as peak oxygen uptake, in young (Milanović et al., 2015) and older adults (Bouaziz et al., 2020). Remarkably, a meta-analysis suggested that exercise programs with a short exercise duration (≤30 min) and a frequency of at least three sessions per week elicited beneficial effects on cognitive performance in older people with and without cognitive impairment (Sanders et al., 2019). This is consistent with existing recommendations for disease prevention and treatment in older adults, which propose a duration of 20–60 min and a frequency of 3–7 days per week for aerobic exercise sessions (Singh, 2002; Izquierdo et al., 2021).

Therefore, the present study investigated the effects of a 30-min IHHE applied prior to a 20-min aerobic cycling exercise on cognitive and physical performance in geriatric patients. We hypothesized that IHHE combined with aerobic cycling training performed three times a week over 6 weeks would have a greater impact on cognitive (operationalized by DemTect and CDT) and physical performance (operationalized by Timed “Up and Go” Test [TUG] and Short-Physical-Performance-Battery [SPPB]) in elderly geriatric patients than aerobic cycling training alone.

## 2 Materials and Methods

### 2.1 Study Design

**FIGURE 1:**
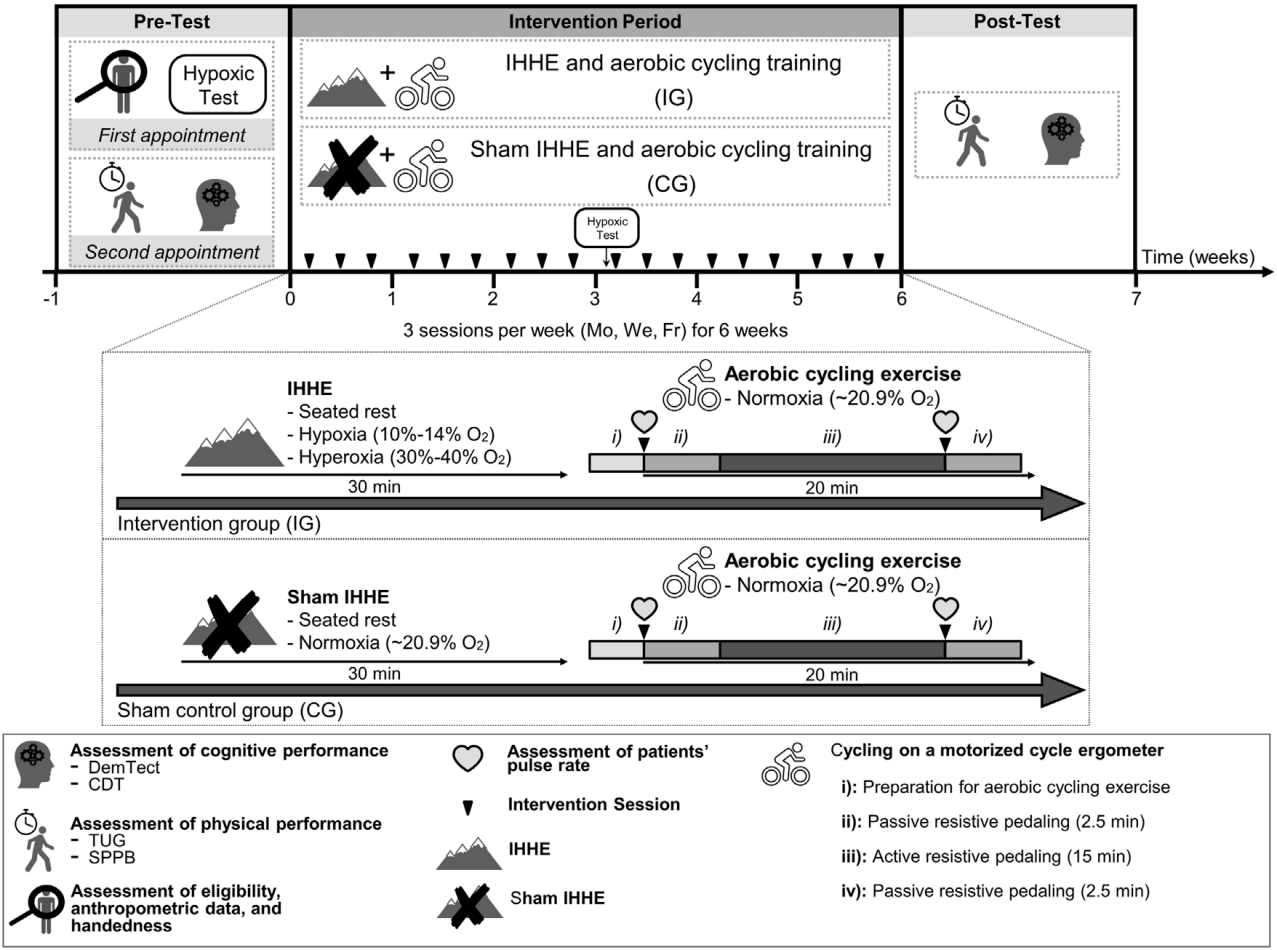
Study design including pre-test (1 week), intervention period (6 weeks), as well as post-test (1 week), and visual presentation of the IG and CG treatment. CDT, clock drawing test; CG, sham control group; DemTect, dementia detection test; Fr, friday; IG, intervention group; IHHE, intermittent hypoxia-hyperoxia exposure; Mo, monday; SPPB, short-physical-performance-battery; TUG, timed “up and go” test; We, wednesday.

**FIGURE 2:**
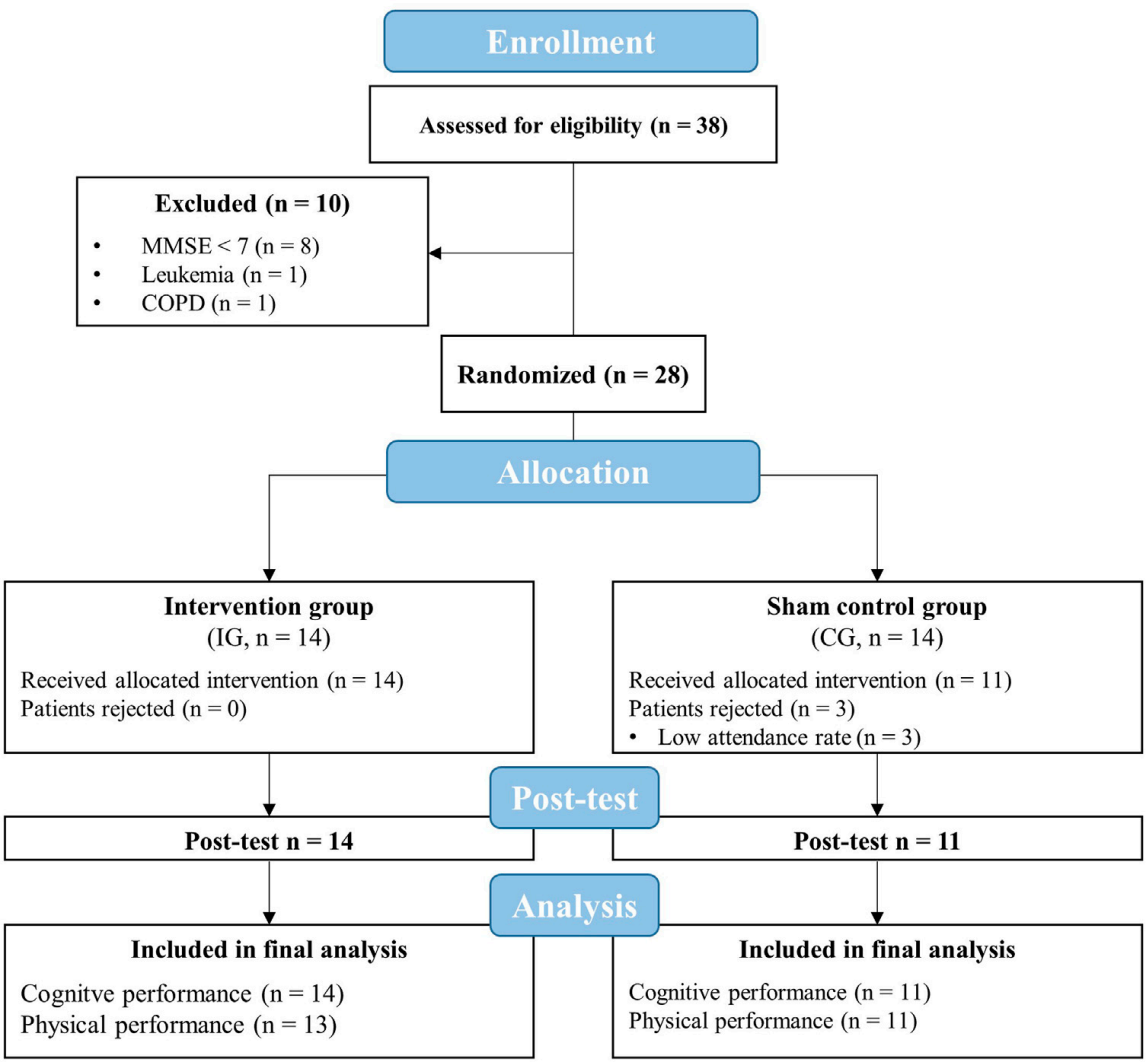
Flow chart of the study. CG, sham control group; COPD, chronic obstructive pulmonary disease; IG, intervention group; MMSE, mini-mental state examination.

This randomized, two-armed, controlled, single-blinded trial was conducted in two inpatient care facilities for geriatric patients*.* Due to a limited number of training devices, the study was carried out in two separated consecutive blocks. Each block lasted 8 weeks consisting of a 1-week pre-diagnostic period, a 6-week intervention period, and a 1-week post-diagnostic period (Figure 1). Prior to the start of the study, patients were informed about the aims and the experimental procedure. After the patients provided their written informed consent, they were enrolled for the pre-diagnostic period consisting of two appointments. During the first appointment, patients’ eligibility to participate, anthropometric data, and handedness [*via* Edinburgh Handedness Inventory (Oldfield, 1971)] were determined. Subsequently, every patient underwent a 10-min lasting hypoxic test (see Section 2.4). During the second appointment, the patients’ level of cognitive impairment was assessed using the Mini-Mental State Examination (MMSE). Further, the DemTect and CDT were conducted to assess patients’ cognitive performance. Additionally, the patients performed the TUG and SPPB to determine their physical performance. All tests were conducted between 1.00 and 5.00 p.m. Following the pre-diagnostic period, patients were randomly assigned to either an intervention group (IG) or a sham control group (CG) using stratified and counterbalanced randomization (randomization was stratified by the MMSE score, allocation ratio was 1:1) by a computer-generated (used software: DatInf Randlist v. 1.5, DatInf GmbH Tübingen, Germany) table of random numbers (Figure 2). The patients were blinded with regard to their group assignment. After 3 weeks of the intervention, patients repeated the hypoxia test (i.e., immediately prior the 10th training session) to determine and adjust the dose of the IHHE. After termination of the intervention period post-tests were performed assessing the patients cognitive and physical performance again.

The study was approved by the Ethics Committee of the Otto-von-Guericke University Magdeburg (No. 202/20) confirming to the principles of the Declaration of Helsinki on human experimentation. The study was retrospectively registered at drks.de (DRKS-ID: DRKS00025130).

### 2.2 Participants

A sample size calculation was performed for a 2 × 2 repeated measures ANOVA using software package G*Power (version 3.1) (Faul et al., 2007). Performance in the DemTect was chosen as primary outcome parameter. However, there are no studies on the comparison of IHHE and sham-IHHE in addition to aerobic exercise, making it impossible to adequately calculate sample size on the basis of preliminary results. Evidence from previous research suggests that there is a moderate effect for the improvement of cognitive performance (i.e., Stroop task performance) in older healthy adults after performing (4 weeks of intervention, three sessions per week) continuous hypoxic exposure (90 min, peripheral oxygen saturation [S_p_O_2_] = 90%-80%) prior to aerobic exercise (20 min, 65%–75% of subjects’ maximum heart rate) compared to a sham control group, conducting aerobic training only (Schega et al., 2016). Thus, a medium effect size (Cohen’s f = 0.25) with a significance level of 0.05, a power of 0.80, and an expected correlation between measures of 0.7 was assumed. According to this, a total sample size of 22 patients (11 patients per group) was required. Considering a drop-out rate of 20%, 28 patients had to be recruited.

Thirty-eight geriatric patients aged over 60 years were screened for eligibility to participate in this study. Exclusion criteria were defined as follows: current smoker, a score of MMSE less than seven points, untreated hypertension, coronary disease with unstable angina pectoris (CCS 3-4), severe heart failure (NYHA III-IV), arrhythmia, untreated and uncontrolled diabetes mellitus, pulmonary fibrosis, chronic obstructive pulmonary disease, cancer, acute inflammatory disease, need for continuous or intermittent ventilation or oxygenation, arterial oxygenation saturation at rest of less than 93%, simultaneously participation in other interventions. After excluding 10 patients (8 patients with MMSE < 7, one patient with leukemia, and one patient with chronic obstructive pulmonary disease), a final number of 28 patients participated in this study (Figure 2).

### 2.3 Intervention

Both groups trained three times per week (Monday, Wednesday, and Friday) for 6 weeks with a total of 18 training sessions. Due to a limited number of training devices, a maximum of four patients could exercise at the same time. Thus, the patients from the same facility were divided into several encounter groups that trained at different times (first block: 9.00–10.00 a.m., 10.30–11.30 a.m., 2.00–3.00 p.m., and 3.30–4.30 p.m.; second block: 8.30–9.30 a.m., 9.45–10.45 a.m., 11.00–12.00 a.m., 1.30–2.30 p.m., and 2.45–3.45 p.m.). Care was taken during group assignment to ensure that the patients from IG and CG were mixed in the encounter groups. Prior to the intervention period, patients were asked to visit a familiarization session to get accustomed to the environment and to estimate patients’ individual level of resistance for the active resistive pedaling phase. For the latter, patients were asked to actively pedal on the motorized cycle ergometer starting with the lowest possible resistance level, which was gradually increased afterwards. At each increment, patients had to indicate whether they thought that the resistance level was appropriate (too easy, too high, and just right) (Holthoff et al., 2015). The program was designed to include patients with obvious cognitive impairment (e.g., dementia). Unfortunately, it was not possible to evaluate the level of effort using a numeric scale such as the CR-10 scale (Griep et al., 1998) or Borg-scale (Borg, 1982) because some patients did not comprehend its meaning.

During each training session, patients of both groups were continuously monitored and supervised by physicians. Each training session lasted approximately 60 min and consisted of two parts.

During the first part, patients of the IG were breathing intermittent hypoxic-hyperoxic air, while those of the CG were breathing normoxic air through a face mask, which was connected to an altitude breathing therapy device (ReOxy, Ai Mediq S.A., Luxembourg). Both procedures (i.e., IHHE and sham IHHE) were applied to the patients while sitting on an armchair for 30 min. In line with current recommendations (Glazachev, 2013; Soo et al., 2020), the hypoxic dose of the IHHE program (i.e., the intensity and duration of a single hypoxic and hyperoxic period) was individually tailored based on the results of the hypoxic test (Section 2.4). The hypoxic test was initially performed during the pre-diagnostic period and was repeated after 3 weeks of the intervention (i.e., immediately prior the 10th training session) in order to re-adjust the hypoxic dose. Furthermore, to control and adjust the hypoxic dose calculated by the hypoxic test (i.e., patients individually tailored minimum S_p_O_2_) during the IHHE session, patients’ S_p_O_2_ and pulse rate were constantly monitored using a fingertip pulse oximeter (Masimo SET, Switzerland) and transmitted to a monitoring device. Thus, the IHHE sessions were tailored to the patients’ individual responses to the hypoxic and hyperoxic exposure by the therapeutic ReOxy device via biofeedback (i.e., when the patient reached the individual minimum S_p_O_2_ or the maximum duration of the hypoxic period estimated by the hypoxic test, the device immediately switched from hypoxic air to hyperoxic air until the initial S_p_O_2_ was recovered). Based on the results of the hypoxic test and the biofeedback, patients from the IG have inhaled hypoxic air (F_i_O_2_ = 0.10–0.14) for 1–5 min, interspersed by a 1–3 min exposure to hyperoxic air (F_i_O_2_ = 0.30–0.40). The intermittent alternation between hypoxic and hyperoxic periods (hypoxic-hyperoxic cycle) was repeated 4–8 times throughout a single 30-min session. The minimum and maximum S_p_O_2_ during the hypoxic periods of the IG was on average about 84 ± 3.4% and 97 ± 1.8%, respectively. The CG received continuous atmospheric normoxic air (F_i_O_2_ ∼0.21) for 30 min and reached an average minimum and maximum S_p_O_2_ of 94 ± 1.9% and 95 ± 1.8%, respectively. The patients were blinded to the air mixture they were breathing.

The second part consisted of supervised 20-min aerobic cycling exercise using a motorized cycle ergometer (MOTOmed viva 2 and viva 1, Reck, Germany). Both the IG and CG performed the training under normoxic conditions without breathing through a facemask. The training program consisting of active and passive phases was designed based on previous studies (Yang et al., 2014; Holthoff et al., 2015) and included: 1) Preparation: The patients sat on an armchair with the cycle ergometer positioned in front of them. The patients’ feet were fixed and the pulse rate of the patients was measured *via* a fingertip pulse oximeter (Pulox PO-300, Novidion GmbH, Germany) and was documented by a therapist. 2) Passive resistive pedaling: The legs of the patients were passively moved by the cycle ergometer for 2.5 min with a constant speed of 20 rpm. 3) Active resistive pedaling: The patients were asked to actively pedale for 15 min at a constant speed of 30–60 rpm. When the patients quit the training program or were pedaling too slow, they were encouraged by a therapist to increase the effort to continue. 4) Passive resistive pedaling: Following the active pedaling, the pulse rate of the patients was again measured *via* a fingertip pulse oximeter and was documented by a therapist. Finally, the patients’ legs were passively moved by the movement trainer at a constant speed of 20 rpm for 2.5 min. The work (kJ) done by the patients was documented for each training session.

### 2.4 Hypoxic Test

Every patient underwent a 10-min lasting hypoxic test (Glazachev, 2013), which consisted of continuously breathing hypoxic air (F_i_O_2_ = 0.12) through a face mask. The hypoxic air mixture was automatically delivered by the altitude breathing therapy device (ReOxy, Ai Mediq S.A., Luxembourg) while the patients were seated on an armchair. During the hypoxic test, patients’ S_p_O_2_ and pulse rate were steadily monitored and stored by the device. Hence, the time to reach the target reduction in S_p_O_2_ (i.e., 80%) was measured as well as the time required to reach the baseline S_p_O_2_. Based on these temporal parameters, the device automatically calculated and recommended individually tailored variables for the IHHE program (i.e., F_i_O_2_, duration of a single hypoxic period, duration of single hyperoxic period, patients’ minimum S_p_O_2_). If the patients’ S_p_O_2_ did not decrease to 80% within 10 min of the test, the duration of the hypoxic phase of the IHHE program was limited to 8 min. In addition, during the hypoxic test, patients’ electrocardiogram was continuously registered by a 12-channel electrocardiogram (cardio 100, custo med GmbH, Germany) and monitored by a physician.

### 2.5 Outcome Measures

Patients’ cognitive status was assessed using the MMSE score to evaluate the severity of cognitive impairment and to screen for patients with a score < 7 (exclusion criteria). The MMSE (Folstein et al., 1975) is a widely used valid and reliable (Tombaugh and McIntyre, 1992) survey designed to screen symptoms of cognitive impairment seen in a variety of dementia conditions. The survey consisted of two parts. The first part requires vocal responses only and includes tests for orientation, memory, and attention performance. The second part assesses the ability to name, follow verbal and written commands, write a spontaneous sentence as well as visual-spatial skills. The maximum total score is 30.

#### 2.5.1 Assessment of Cognitive Performance

First, cognitive performance of the patients was assessed using the DemTect and CDT. Previous studies have shown that the DemTect and the CDT can be used as a sensitive tool to evaluate the effects of an intervention on changes in cognitive performance in older geriatric patients (Bayer et al., 2017a; Bayer et al., 2017b). The patients sat at a table in front of the investigator in a quiet room. No clock was visible to the patient during the test.

The DemTect (Kalbe et al., 2004) is a sensitive screening tool to detect mild cognitive impairment and early stages of dementia. It consists of five tasks that have been proven to be highly sensitive in the diagnosis of dementia: 1) verbal memory task (word list), 2) number transcoding task, 3) semantic verbal fluency task, 4) digit span reverse task, and 5) delayed recall task (world list). The maximum total raw score is 70. The raw scores for each task were transformed using a transformation table (Kessler et al., 2014). The transformed scores are age corrected and the subtests are weighted according to their individual sensitivity and specificity. The maximum total transformed score is 18 with higher scores associated with better global cognitive function.

After termination of the DemTect, the CDT was performed. Originally, the CDT was developed and used to assess visuo-constructive abilities (Freedman et al., 1994). However, the test is also used as a reliable test to screen for cognitive impairment and dementia as well as for spatial dysfunction and neglect (Agrell and Dehlin, 1998). For the test procedure, each patient was given a blank sheet and a ballpoint. Subsequently, patients were asked to follow a two-stepped instruction: 1) First, the patients were asked to draw a clock with all the numbers on the sheet without drawing in the clock hands. 2) After completing this task, the patients were asked to add the clock hands to make it read 2:45. The instruction was repeated as often as necessary, but no other hints were given. Three raters independently and blindly evaluated the CDT based on the Sunderland scoring system (Sunderland et al., 1989) (from 10 “best representation of a clock” to 1 “worst representation of a clock”). This scoring system is highly specific and predictive to determine dementia, making it valuable when using the CDT in clinical purpose (Park et al., 2018). Any case of disagreement in the rating scores were resolved by discussion and re-evaluation.

#### 2.5.2 Assessment of Physical Performance

The examinations to determine patients’ physical performance were performed in a fixed order following the CDT. First, patients performed the TUG (Podsiadlo and Richardson, 1991), which is a reliable (Ries et al., 2009) and valid (Brooks et al., 2006) test for assessing functional mobility. The TUG quantified the time required for the patients to get up from a chair with armrests, walk 3 m, turn 180°, walk back to the chair, and sit down again. In the present study, the patients walked around a cone placed at the 3 m mark and were instructed to walk fast but safely. If the patients used a walking aid in everyday life, they were asked to also use this walking aid to perform the TUG. The time started when the patients’ back left the backrest of the chair and stopped when the bottom touched the chair again after walking. The best of two attempts was scored. Identical chairs were used for the pre- and post-test.

Subsequently, the patients were asked to perform the SPPB (Guralnik et al., 1994), which consisted of three subtests. First, patients’ standing balance including side by side (heel and toe of one foot were placed to the side of the heel and toe of the other foot), semi-tandem (heel of one foot was placed to the side of the toe of the other foot), and tandem stand (heel of one foot directly in front of the other foot) were determined. The SPPB is an objective, reliable, valid, and promising tool to evaluate the physical performance level in patients with different disease (Ortega-Pérez de Villar et al., 2018). Each patient began with the semi-tandem stand. Those patients able to hold the semi-tandem stand for 10 s were further evaluated with the feet in tandem stand. Otherwise, they performed the side by side stand. For each standing position, a therapist demonstrated the task and, subsequently, supported one arm while the patients positioned their feet. If the patients were ready, the therapist released the support and started timing. Time was stopped when patients moved their feet, asked the therapist for assistance, or 10 s had elapsed. Second, a 4-m walk at patients’ habitual pace was timed and scored according to the length of time required. A 5-m walking course, with no barriers for additional 2 m at the end, was marked and the patients were instructed to walk with their habitual speed to the end of this course. A cone was placed at 4 m so that the therapist could stop the time when the patient reached the 4-m mark. Patients were allowed to use walking aids when needed. The time of the fastest of the two walking trials was recorded and used for scoring. Third, patients’ ability to stand up from a chair without using their hands was assessed (also known as chair-rising-test or sit-to-stand-test). Patients were asked to stand up from a chair once while crossing their arms in front of their chest. If they were successfully risen up from the chair, they were asked to stand up and sit down five times as fast as possible. The time started when the patients’ bottom left the chair and was stopped at the final stand position (at the end of the fifth stand). The results of each subtest were scored according to predefined quantiles. The total SPPB score was determined by adding the subscores of the standing-balance-test, the 4-m walk-test, and the sit-to-stand-test, with each subscore ranging from 0–4, giving a maximum total score of 12 points. A score of 0 represents the worst possible performance and a score of 12 the best possible performance.

Both the TUG and the SPPB can be used to determine performance changes over time and to assess the efficiency of interventions to maintain or improve functional mobility or physical performance in older geriatric patients (Ries et al., 2009; Ortega-Pérez de Villar et al., 2018). Studies investigating the absolute reliability of these tests have found a minimal detectable change (MDC) of 4.09 s and 0.8 points for the TUG and SPPB, respectively (Ries et al., 2009; Olsen and Bergland, 2017). These MDC values can be used to assess whether changes in physical performance after an intervention are due to a real change and a measurement error. For the present study, this implies that a difference of 4.09 s or 0.8 points in the TUG or SPPB, respectively, would be sufficient to assume that the measurement error was exceeded.

### 2.6 Statistical Analysis

Data analyses were conducted using SPSS Statistics 26 (IBM, Inc., Chicago). Data were tested for normal distribution and homogeneity of variance using the Kolmogorov-Smirnov-Test and Leven’s test, respectively. For normally distributed data, the mean and standard deviation and for non-normally distributed data the median and the 25th and 75th percentile are reported. As the primary analysis, a 2 (time: pre-test, post-test) × 2 (group: IG, CG) repeated measures ANOVA was performed to check for interaction effects and main effects. If baseline values differed between groups, univariate ANCOVA with baseline values entered as a covariate (baseline-adjustment) was performed. Differences between pre- and post-test values are presented as means (± standard deviations) and as the percentage change, if feasible. Furthermore, 95% confidence intervals (CI) are shown to provide information about the magnitude and direction of an effect (Page, 2014). Unpaired *t*-tests and paired *t*-tests were used to check the data for differences between groups and between the pre- and post-test within a group, respectively. The data of the DemTect and the CDT did not show variance homogeneity and normal distribution, respectively. Since it was shown that the ANOVA (Schmider et al., 2010; Blanca et al., 2017) and *t*-test (Havlicek and Peterson, 1974; Pagano, 2009) could be used without significant error despite moderate violation of homogeneity or normality assumptions, no alternative nonparametric tests were performed. Subgroup analysis was conducted according to the level of cognitive impairment at baseline by performing a three-way ANOVA (level of cognitive impairment × group × time). The level of cognitive impairment was dichotomized based on the median MMSE score of 18 (≤18 and ≥19).

It is recommended to use the effect size for interpreting the results of interventional studies and clarifying the practical and clinical relevance of the results (Lakens, 2013). Thus, the effect sizes partial eta squared (*η*_*p*_^*2*^) and Cohen’s d (*d*) were calculated. The *η*_*p*_^*2*^ used for the ANOVA and ANCOVA was classified as 0.01 = small, 0.06 = medium, and 0.14 = large effect (Cohen, 2013; Lakens, 2013). The effect size Cohen’s d was used to determine the relevance of mean differences between two groups or time-points for the unpaired and paired *t*-tests, respectively, with 0.20–0.49 indicating a small, 0.50–0.79 indicating a medium, and >0.80 indicating a large effect (Cohen, 1992, Cohen, 2013). Effects sizes were calculated using the following formula: *η*_*p*_^*2*^ = $\frac{F\times df_{effect}}{F\times df_{effect}+df_{error}}$ , $d\left(unpaired\;t-test\right)=t\times \sqrt{\frac{1}{n_{1}}+\frac{1}{n_{2}}}$ , and $d\left(paired\;t-test\right)=\frac{t\;}{\sqrt{n}}$ .

## 3 Results

### 3.1 Participants and Training

Out of 28 included patients, three patients from the CG had to be excluded from the final analysis because of their low attendance rate (<80%). The remaining 25 patients (IG = 14, CG = 11) had a sufficient attendance rate during the intervention period (IG = 98 ± 3%, CG = 95 ± 6%) and were included in the data analysis. Thus, the planned sample size (at least 11 patients per group) was achieved. The TUG and SPPB of one patient in the IG could not be recorded at the post-test due to an acute illness (rheumatoid arthritis relapse). The intermittent hypoxic-hyperoxic exposures as well as the aerobic cycling training were well tolerated. There were no injuries or adverse side effects except some reports of mild dizziness.

No significant differences in the patients’ demographic characteristics (age, height, weight, and body mass index) and MMSE scores were found between IG and CG (Table 1). The minimum S_p_O_2_ values shown in Table 1 were lower in the IG during the IHHE than in the CG during the sham IHHE (*T*_*23*_ = 10.038, *p* < 0.001, *d* = 4.04). There were no significant differences between groups with respect to the average work (kJ) performed during the 20 min cycling training (*T*_*23*_ = 0.318, *p* = 0.753, *d* = 0.13) as well as the average pulse rate at the beginning (*T*_*23*_ = 0.602, *p* = 0.553, *d* = 0.24) and the end (*T*_*23*_ = 0.446, *p* = 0.660, *d* = 0.18s.o.) of the cycling training sessions (Table 1). To be able to compare the intensity of the aerobic training of this study with those used by other studies, the percentage of the estimated maximum heart rate (208−0.7 × age (Tanaka et al., 2001)) was retrospectively calculated. The IG and CG patients achieved 51 ± 6% (range 40%–62%) and 52 ± 7% (range 42%–63%), respectively, of their estimated maximal heart rate at the end of each aerobic exercise session.

**TABLE 1:** Demographic and clinical characteristics of the patients at baseline as well as description of the IHHE, sham IHHE, and aerobic cycling training sessions.

Characteristics	IG (*n* = 14)	CG (*n* = 11)
Age [years]	84.2 ± 5.1	85.6 ± 6.0
Female [n (%)]	13 (93%)	11 (100%)
Height [cm]	159.9 ± 8.1	156.4 ± 10.9
Weight [kg]	71.2 ± 11.9	65.6 ± 13.0
Body mass index [cm/kg^2^]	27.8 ± 4.1	26.7 ± 3.9
Mini-mental state examination [score]	18.6 (7.2)	17.7 (6.5)
>24 [n (%)]	4 (29%)	2 (18%)
24-21 [n (%)]	2 (14%)	2 (18%)
20-11 [n (%)]	6 (43%)	6 (55%)
10-7 [n (%)]	2 (14%)	1 (9%)
Clinical diagnosis [n (%)]		
Alzheimer’s dementia	3 (21%)	5 (45%)
Vascular dementia	0 (0%)	1 (9%)
Unspecified dementia	5 (36%)	4 (36%)
Hypertension	11 (79%)	10 (91%)
Diabetes mellitus type II	4 (29%)	4 (36%)
Hyperlipidaemia	2 (14%)	2 (18%)
Hypercholesterinaemia	1 (7%)	2 (18%)
Hyperthyreosis	0 (0%)	3 (27%)
Hypothyreosis	2 (14%)	2 (18%)
Osteoporosis	2 (14%)	5 (45%)
Regular medications [n (%)]		
ACE inhibitors	4 (29%)	3 (27%)
AT1 receptor antagonist	5 (36%)	4 (36%)
β-blocker	5 (36%)	7 (64%)
Calcium channel blocker	4 (29%)	1 (9%)
Diuretics	5 (36%)	1 (9%)
Acetylsalicylic acid	5 (36%)	4 (36%)
Heparinoid	1 (7%)	1 (9%)
Statins	2 (14%)	0 (0%)
L-thyroxin	2 (14%)	3 (27%)
Metformin	2 (14%)	1 (9%)
Insulin	1 (7%)	1 (9%)
Melperon	3 (21%)	3 (27%)
Antidepressant agents	2 (14%)	4 (36%)
Galantamin	2 (14%)	1 (9%)
Memantin	1 (7%)	3 (27%)
Donepezil	0 (0%)	2 (18%)
Average minimum and maximum S_p_O_2_ recorded during the IHHE or sham-IHHE sessions		
Minimum S_p_O_2_ [%]	84.10 ± 2.61	93.55 ± 1.92
Maximum S_p_O_2_ [%]	96.83 ± 1.84	95.89 ± 1.80
Variables recorded before, during, and after the aerobic cycling training sessions		
Pulse rate before exercise [beats·min^−1^]	73.79 ± 2.84	76.25 ± 2.88
Work [kJ]	87.03 ± 10.90	93.80 ± 19.66
Pulse rate after exercise [beats·min^−1^] (% of estimated maximum heart rate)	75.31 ± 2.53 (51% ± 6%)	77.15 ± 3.34 (52% ± 7%)

CG, sham control group; IG, intervention group.

To determine the practical relevance and generalizability of the effect of IHHE prior to aerobic training on cognitive and physical performance in geriatric patients, results were interpreted based on effect sizes (Lakens, 2013).

### 3.2 Cognitive Performance

**FIGURE 3:**
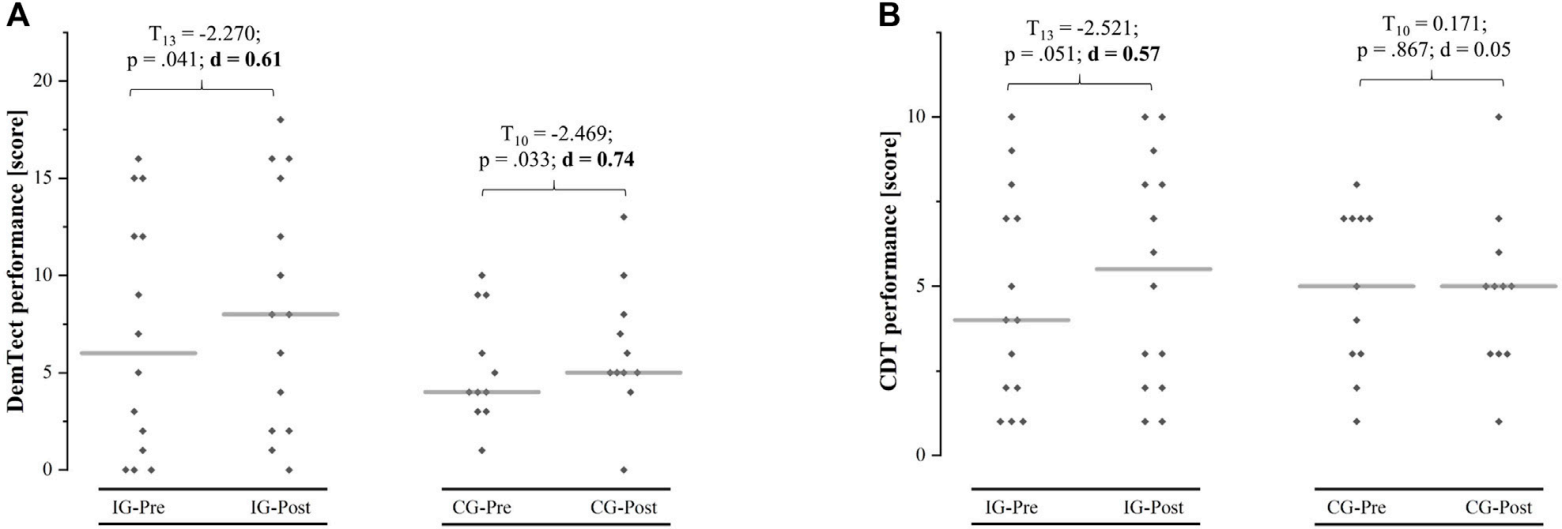
Mean (grey line) and individual (grey squares) values of the dementia detection test (DemTect) and clock drawing test (CDT) performance at pre- and post-test for the intervention group (IG) and the sham control group (CG).

Before the intervention, there were no differences between groups regarding the DemTect (*T*_*24*_ = 0.822, *p* = 0.384, *d* = 0.33) and CDT (*T*_*24*_ = −1.080, *p* = 0.261, *d* = 0.44). No interaction effect or main effect of group was found with respect to the DemTect. However, a main effect of time with a large effect size was found, indicating an improved DemTect performance after both interventions (IG: 6.9 ± 6.1–8.4 ± 6.2; CG: 5.3 ± 2.9–6.2 ± 3.4). An interaction effect with a medium effect size was found for CDT performance with a higher change from pre- to post-test for the IG (4.0 [1.8/7.3]–5.5 [2.0/8.3]; +28%) compared to CG (5.0 [3.0/7.0]–5.0 [3.0/6.0]; +4%). Table 2 shows the mean differences (± standard deviations) from pre- to post-test, the 95% confidence interval, and the results of the 2 × 2 repeated measure ANOVA (F-value, df_effect_, df_error_, *p*-value, and effect size η_p_^2^). Cognitive performance measures obtained at the pre- and post-test as well as the results of the post-hoc analysis (*t*-value, df, *p*-value, and effect size d) are depicted in Figure 3. Subgroup analysis taking the level of cognitive impairment into account (MMSE score) did not reveal interaction effects for DemTect (*F*_*1,21*_ = 0.003, *p* = 0.958, *η*_*p*_^*2*^ = 0.00) or CDT (*F*_*1,21*_ = 0.076, *p* = 0.786, *η*_*p*_^*2*^ = 0.00) (Supplementary Table S1).

**TABLE 2:** Pre-post results of the dementia detection test (DemTect) and clock drawing test (CDT).

Measure	Group	MD ± SD	CI (95%)	Interaction effect	Main effect of time	Main effect of group
DemTect [score]	IG	1.50 ± 2.47	0.07–2.93	*F*_*1,23*_ = 0.524, *p* = 0.476, *η*_*p*_^*2*^ = 0.02	*F*_*1,23*_ = 8.710, *p* = 0.007, *η*_*p*_^*2*^ = 0.28	*F*_*1,23*_ = 0.946, *p* = 0.341, *η*_*p*_^*2*^ = 0.04
	CG	0.91 ± 1.22	0.09–1.37			
CDT[score]	IG	0.79 ± 1.37	0.00–1.58	*F*_*1,23*_ = 1.970, *p* = 0.174, *η*_*p*_^*2*^ = 0.08	*F*_*1,23*_ = 1.238, *p* = 0.277, *η*_*p*_^*2*^ = 0.05	*F*_*1,23*_ = 0.008, *p* = 0.930, *η*_*p*_^*2*^ < 0.01
	CG	−0.09 ± 1.76	−1.27–1.09			

CDT, clock drawing test; CG, sham control group; CI, confidence interval; DemTect, dementia detection test; IG, intervention group; MD, mean difference; SD, standard deviation.

### 3.3 Physical Performance

**FIGURE 4:**
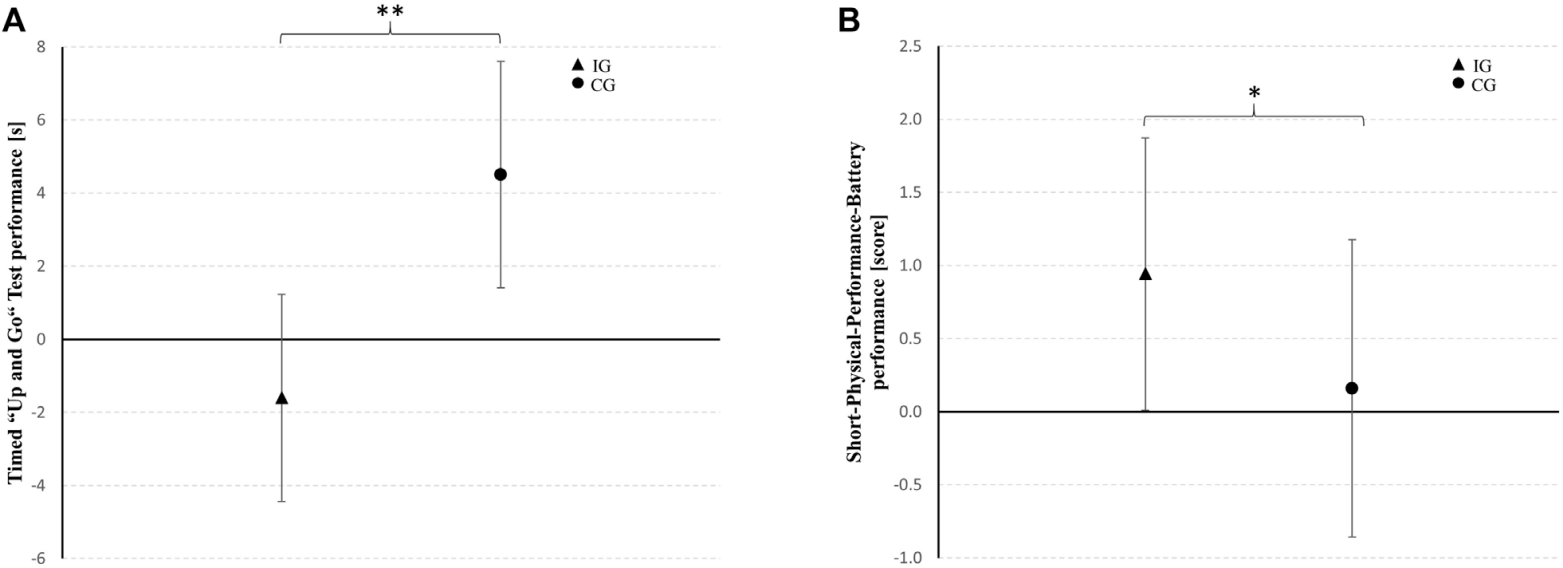
Between-group differences from baseline in the timed “up and go” test and short-physical-performance-battery performance for the intervention group (IG) and the sham control group (CG) represented as mean changes with 95% confidence intervals. * medium effect (*η*_*p*_^*2*^ = 0.06–0.13); ** large effect (*η*_*p*_^*2*^ ≥ 0.14).

At pre-test, differences were found between IG and CG in TUG performance (*T*_*23*_ = −2.126, *p* = 0.044, *d* = 0.86) and SPPB performance (*T*_*23*_ = 1.950, *p* = 0.063, *d* = 0.79). Therefore, univariate ANCOVAs with pre-test values entered as covariate were performed. The ANCOVA with baseline-adjustment showed differences between groups at post-test for the TUG and SPPB performance in favor of the IG with large and medium effect sizes, respectively. Within-group post-hoc analysis showed that the time needed to perform the TUG was increased in the CG (7%; *T*_*10*_ = −2.164, *p* = 0.056, *d* = 0.65). In contrast, the patients of the IG revealed no change over time in TUG performance (−6%; *T*_*12*_ = 0.706, *p* = 0.493, *d* = 0.19). Furthermore, SPPB performance was improved in the IG (+72%; *T*_*12*_ = −2.156, *p* = 0.052, *d* = 0.58) and remained unchanged in the CG (−1%; *T*_*10*_ = 0.000, *p* = 1.000, *d* = 0.00) from pre- to post-test. Table 3 shows the physical performance outcomes at pre-test as well as the baseline-adjusted mean values and standard deviations at post-test together with the adjusted mean differences (95% confidence interval) between IG and CG at post-test and the results of the univariate ANCOVA (F-value, df_effect_, df_error_, *p*-value, and effect size *η*_*p*_^*2*^). Between group-differences from baseline in TUG and SPPB performance for the IG and CG are shown in Figure 4. The subgroup analysis indicated that the patients’ level of cognitive impairment did not affect the intervention effects on TUG (*F*_*1,20*_ = 0.007, *p* = 0.936, *η*_*p*_^*2*^ = 0.00) and SPPB (*F*_*1,20*_ = 0.022, *p* = 0.884, *η*_*p*_^*2*^ = 0.00) (Supplementary Table S1).

**TABLE 3:** Pre-test and baseline-adjusted post-test values as well as mean differences [95% confidence interval (CI)] for the timed “up and go” test (TUG) and short-physical-performance-battery (SPPB).

Variable	Pre-test		Post-test^a^			ANCOVA
	IG	CG	IG	CG	MD_(IG-CG)_ (95% CI)	
TUG [s]	23.2 ± 11.4	15.2 ± 5.5	17.2 ± 5.1	23.3 ± 4.9	−6.1 (−10.5–−1.8)	*F*_*1,21*_ = 8.558, *p* = 0.008, *η*_*p*_^*2*^ = 0.29
SPPB [score]	4.6 ± 2.3	6.5 ± 2.6	6.4 ± 1.7	5.7 ± 1.6	0.8 (−0.6–2.2)	*F*_*1,21*_ = 1.304, *p* = 0.266, *η*_*p*_^*2*^ = 0.06

a Data are presented as baseline-adjusted means ± standard deviations and adjusted mean differences (95% confidence interval) between IG and CG at post-test (IG-CG). CG, sham control group; IG, intervention group; MD, mean difference.

## 4 Discussion

The purpose of the present study was to investigate the effects of an IHHE applied prior to an aerobic exercise on cognitive and physical performance in geriatric patients. The results indicate that IHHE combined with subsequent aerobic cycling exercise performed for 6 weeks seemed to be more effective to 1) increase global cognitive functions (increased CDT performance) and 2) physical performance (increased SPPB performance) in geriatric patients in comparison to aerobic cycling training alone. Moreover, 3) a preserved functional mobility (maintained TUG performance) was found in those patients who performed IHHE prior to aerobic cycling exercise after the intervention period. Subgroup analysis indicated that the intervention effects did not differ when taking the patients’ level of cognitive impairment at baseline into account. The experiences gained in the study suggest that IHHE is applicable and well tolerated as an adjunct to aerobic training in elderly geriatric patients with and without cognitive impairment.

### 4.1 Effects on Cognitive Performance

There is accumulating evidence supporting the notion that physical training (e.g., aerobic training) induces positive effects on cognitive performance in older adults (Bouaziz et al., 2017; Northey et al., 2018). However, the effect of well-dosed hypoxia on cognitive performance in the elderly has not been studied as thoroughly (irrespective whether hypoxia-normoxia (Schega et al., 2013; Schega et al., 2016; Wang et al., 2020) or hypoxic-hyperoxic intervals (Bayer et al., 2017a; Serebrovska Z et al., 2019) were used). The results of the present study show that patients, which received IHHE prior to aerobic exercise improved their performance in both cognitive tests, the DemTect and the CDT, after the intervention. However, the beneficial effect of an additional IHHE prior to aerobic cycling exercise on cognitive performance could only be demonstrated for the CDT, because the patients not breathing IHHE prior to aerobic cycling have also improved in DemTect similar to the patients which were given IHHE.

Beside the type of exercise that is used for the training intervention (e.g., aerobic and/or resistance exercise), general exercise variables (e.g., intensity and duration of a single exercise session), and training variables (e.g., frequency and total intervention duration), the relationship between physical training and cognitive performance has been shown to be influenced by the cognitive domain or ability (e.g., executive function and memory) assessed by the used specific cognitive test (Zheng et al., 2016; Northey et al., 2018; Herold et al., 2019; Pontifex et al., 2019; Huang et al., 2021). In this regard, cognitive tests could be categorized according to the specific cognitive ability they purport to quantify (Pontifex et al., 2019). However, global cognitive tests (e.g., the DemTect or CDT) are intended to capture multiple cognitive abilities (Riello et al., 2021). Thus, it can be inferred that several distinct cognitive abilities are required for the completion of the DemTect and CDT. The five subtests of the DemTect cover a wide range of cognitive abilities including immediate and delayed recall of verbal information, language and number processing, working memory, and executive functioning (Kalbe et al., 2004). On the other hand, completion of the CDT requires a mixture of visuospatial skills and executive control functions, e.g., visual memory and reconstruction in a graphic image, abstract thinking, as well as motor programming and execution (Shulman, 2000). In this regard, meta-analysis have indicated that the effects of aerobic training on these different cognitive ability domains are selective (Smith et al., 2010; Zheng et al., 2016; Sanders et al., 2019). Therefore, it appears that the additional use of IHHE increased the efficacy and/or prolonged the effects of the subsequent aerobic cycling exercise on one or multiple specific cognitive abilities tested by the CDT.

These results are similar to those reported in a previous study investigating the effect of IHHE in addition to physical training on cognitive performance in geriatric patients (Bayer et al., 2017a). Bayer et al. (2017a) demonstrated that IHHE provided in addition to an individualized multimodal training program, which included aerobic, strength, balance, flexibility, coordinative, and cognitive exercises (2–6 times per week, 14-15 sessions in 5–6 weeks) has augmented the effects on global cognitive functions operationalized with the DemTect and CDT in comparison to an individualized multimodal training program alone. However, although the authors reported that the group of geriatric patients who had conducted the additional IHHE exhibited an increased performance in the DemTect (+3 points [+16.7%]) and CDT (+1.07 points [+10.7%]) at the end of the intervention, no changes were observed in those patients who had conducted sham IHHE in addition to an individualized multimodal training program (DemTect: +0.07 points [+0.39%]; CDT: −0.8 points [−8%]). These findings are in contrast to the results of the present study, which indicates that DemTect performance was also increased in those patients who performed aerobic cycling training without an additional IHHE. Bayer et al. (2017a) argued that the lack of improvements in global cognitive performance after their multimodal training program was related to the low physical capacity of the geriatric patients who were older than 80 years and which prevented reaching an sufficient training intensity necessary to achieve detectable neurocognitive effects. However, the patients from the present study and from the study by Bayer et al. (80.9 ± 7.9 years and 83.4 ± 5.5 years) had comparable mean ages. Apart from age, differences in the level of cognitive impairment could explain the discrepant results. In this regard, the mean MMSE score of the patients from the present study was lower compared to those from the study by Bayer et al. (IHHE group: 24.9 ± 3.8, sham IHHE group: 24.5 ± 3.9), indicating more severe cognitive impairment. This is in agreement with the findings of a meta-analysis, showing that the effect of physical training on global cognitive function was higher in patients with cognitive impairment (*d* = 0.37) compared to healthy individuals (*d* = 0.17) (Sanders et al., 2019). However, the exact explanation for the different results remains unclear.

A further pilot study (Serebrovska Z. et al., 2019) that evaluated the effect of solely IHHE on cognitive performance in patients with MCI found an increased total score in the Montreal Cognitive Assessment test which is a measure of global cognitive function (Nasreddine et al., 2005). Furthermore, a downward trend in the P300 and N200 cognitive evoked potentials’ latency along with a decrease in non-stimulated neurotrophic extracellular traps and amyloid-beta expression in peripheral blood were observed after 3 weeks of intervention with five sessions per week (Serebrovska Z et al., 2019). Neurotrophic extracellular trap formation and amyloid-beta accumulation are thought to play a major role in several neurodegenerative processes that gradually lead to Alzheimer’s disease (Palop and Mucke, 2010; Zenaro et al., 2015). In particular, accumulation of amyloid-beta can result, among others, in cerebral amyloid angiopahty-related capillary occlusion, which is associated with cerebral blood flow disturbances (Thal et al., 2009; Korte et al., 2020) and neuronal dysfunctions (Sadigh-Eteghad et al., 2015). Interestingly, a meta-analysis of 216 studies demonstrated that cerebral blood flow in the parietal and temporal lobes as well as in the hippocampus already decreases during the earliest stages of disease (e.g., in patients with mild cognitive impairment) (Zhang et al., 2021). The authors suggest that amyloid-beta deposition is triggered by early-stage cerebral hypoperfusion. The neurotoxic effects of amyloid-beta, in turn, further exacerbate cerebral hypoperfusion due to impaired vascular function, leading to a progressive decline in cognitive performance and brain mass. In a rat model of Alzheimer’s disease induced by administration of amyloid-beta, researchers found that adaptations to hypobaric hypoxia (4 h per day for 14 days) prevented endothelial dysfunction of cerebral vessels and attenuated cerebral blood flow disturbance (Mashina et al., 2006). In this regard, a recent pilot study demonstrated that 8 weeks of intermittent exposure to hypoxia and normoxia (three sessions per week) augmented cerebral oxygenation and cerebrovascular dilation while improving global cognitive function (operationalized by MMSE) and short term memory (operationalized by digital span test) in patients with amnestic MCI (Wang et al., 2020). It is suggested that transcriptional genes such as nitric oxide synthase (responsible for vasodilation, among other things) and vascular endothelial growth factor (responsible for angiogenesis, among other things), which are expressed by hypoxia induced molecular signalling pathways, are intricately involved in cerebral blood flow regulation (Pichiule and LaManna, 2002; Hickey and Simon, 2006; Atochin and Huang, 2011) and thus may be responsible for the beneficial effect of IHHE on cognitive performance reported in the present study. Beside a decreased amyloid-beta expression and improved cerebrovascular function, further neuroprotective mechanisms of intermittent hypoxia-hyperoxia or hypoxia-normoxia, such as reduced vascular risk factors, decreased inflammation and oxidative stress as well as stimulated neurogenesis and neuroregeneration, are summarized and discussed by several authors (Manukhina et al., 2016; Mallet et al., 2020; Burtscher et al., 2021). However, the exact mechanisms are not yet known and the present study did not investigate any of the possible mechanisms, so no further conclusions can be drawn.

### 4.2 Effects on Physical Performance

Even though TUG performance did not improve in both groups, data of the present study suggests that the additional use of IHHE had a superior effect compared with aerobic cycling training alone. This was particularly indicated by the increased time needed to perform the TUG in those patients who received sham IHHE, whereas the patients who received IHHE maintained their TUG performance. Although the TUG is recommended as a routine screening test to detect older adults at an elevated risk of falling (American Geriatric Society and British Geriatric Society, 2011), systematic reviews concluded that its predictive ability for future falls in older adults remains limited (Beauchet et al., 2011; Barry et al., 2014). However, it has been shown that TUG performance is associated, among others, with lower limb muscle strength and power, balance, aerobic capacity, and usual as well as maximal walking velocity in older females, indicating that this test may be a useful tool to evaluate functional mobility in the elderly (Coelho-Junior et al., 2018). Furthermore, the SPPB results showed that the total score increased over time in those patients who conducted the IHHE procedure, while it remained unchanged in those patients who performed aerobic cycling training only. These results suggest that an IHHE prior to an aerobic cycling training could maintain functional mobility and could even improve physical function in geriatric patients, whereas aerobic cycling training alone induced no improvements. In general, aerobic training was shown to be an effective intervention strategy to induce central and peripheral adaptations that improve cardiovascular capacity (e.g., peak oxygen consumption) (Cadore et al., 2014; Bouaziz et al., 2020). These adaptations include an enhanced cardiac output and stroke volume (Murias et al., 2010) as well as an increased mitochondrial biogenesis, capillary density, and oxidative enzyme activity (Meredith et al., 1989; Heinonen et al., 2014). Moreover, aerobic training is considered as an effective strategy to improve functional status (i.e., muscle strength, physical performance, and risk of falling) in adults aged 70 years and older (Bouaziz et al., 2017). Previous studies show that IHHE alone may be effective to increase peak oxygen consumption in cardiology outpatients with diverse comorbidities (Dudnik et al., 2018) and in older patients with coronary heart disease (Glazachev et al., 2017; Glazachev et al., 2019). With respect to passive intermittent hypoxic-normoxic exposure, studies have shown increased exercise tolerance and peak oxygen consumption in elderly, physically active males with New York Heart Association class I and II (Burtscher et al., 2004) and greater positive effects on hemodynamic, microvascular endothelial function, and work capacity in untrained, healthy older males (Shatilo et al., 2008) compared to pre-intervention. Furthermore, Saeed et al. (2012) indicated that brief continuous hypoxic exposure (3–4 h per session) leads to significant increase in peak oxygen consumption, 6-min walk distance, isometric muscle strength (arm flexion), and a trend for a significant increase in left ventricular ejection fraction and diastolic diameter in patients with heart failure and reduced left ventricular ejection fraction. With regard to the present study, the beneficial effect of IHHE on physical performance could be explained by specific cardiovascular and muscular adaptations, such as angiogenesis, improved glycose transport, glycolysis, and pH regulation as well as mitochondrial functioning (Gore et al., 2007; Verges et al., 2015). Contrary, Bayer et al. (2019) did not confirm the hypothesis that IHHE would significantly improve the efficiency of an individualized multimodal training program on functional mobility or physical performance in geriatric patients, operationalized by TUG, Tinetty Mobility Test, and Barthel Index. Interestingly, Bayer et al. (2019) demonstrated improvements in all three tests in both groups, suggesting that the individualized multimodal training program was effective regardless of the addition of IHHE. However, the study did not include a passive control group, thus the results should be interpreted with caution. In another study, the same authors reported higher increases in functional capacity examined with the 6-min walk test in those geriatric patients who performed an IHHE in addition to an individualized multimodal training program (Bayer et al., 2017a). While the physical training in the present study only consisted of aerobic cycling training, the individualized multimodal training program in the studies by Bayer et al. (2019) included various types of training, such as aerobic, strength, balance, flexibility, coordinative, and cognitive training. Previous evidence indicated that different types of training (e.g., strength or aerobic training) affect different aspects of physical function and appears to be an important factor in obtaining beneficial effects on specific outcomes (e.g., functional mobility, physical function or aerobic capacity) (Cadore et al., 2014; Lam et al., 2018). In this regard, the acute and chronic responses to exercise or training (Heinonen et al., 2014) and hypoxia (Lee et al., 2019) are complex and potentially act synergistically (due to similar or unique mechanisms) to improve physical performance. However, little is known about the optimal type and dose of physical exercise or hypoxia and the most favourable combination of these components (Millet et al., 2016).

### 4.3 Dose of IHHE

The effects of intermittent hypoxia have been studied in both directions, as a cause of or as a therapy for various diseases, such as metabolic, cardiovascular, neuronal, and pulmonary disease (Almendros et al., 2014). For instance, the adverse effects of chronic intermittent hypoxia have been frequently studied in patients with obstructive sleep apnea (Gozal and Kheirandish-Gozal, 2008; Arnardottir et al., 2009; Beaudin et al., 2017). In general, obstructive sleep apnea is a chronic, sleep-related breathing disorder caused by complete or partial upper airway obstruction that can lead to various physiological disturbances, e.g., arterial hypoxemia (Maspero et al., 2015). Although other potential risk factors (e.g., hypercapnia and arousal) also exist, chronic intermittent hypoxia is believed to be one of the primary mediators for the development of a number of detrimental cardiovascular, respiratory, metabolic, and cognitive outcomes in patients with obstructive sleep apnea (Arnardottir et al., 2009; Beaudin et al., 2017). However, this is mainly true for patients with severe symptoms. In patients with mild symptoms, the effects are still unclear with one review suggesting that mild obstructive sleep apnea may produce contrasting/beneficial effects (Beaudin et al., 2017).

Generally, it is assumed that the maladaptive or adaptive responses to intermittent hypoxia could be either pathological or beneficial, respectively. This depends, among other factors, on the hypoxic dose (e.g., intensity, duration, and frequency of the hypoxic stimulus). In this regard, a hypoxic dose consisting of a high number of hypoxic episodes per day (high frequency) with a high degree of hypoxemia (F_i_O_2_ = 0.02–0.08) was considered to be associated with adverse effects on different organ systems (Navarrete-Opazo and Mitchell, 2014; Mateika et al., 2015). On the other hand, accumulating evidence suggests that “moderate- to low-dose” intermittent hypoxia protocols with a low frequency (2–15 cycles per day) of relatively brief duration (1–10 min per hypoxic episode), and an intensity/severity of 0.09–0.16 F_i_O_2_ can elicit beneficial therapeutic effects (Navarrete-Opazo and Mitchell, 2014; Serebrovska et al., 2016). However, these recommendations refer to hypoxic episodes (e.g., intermittent hypoxic-normoxic exposure). As with hypoxia, hyperoxia can also elicit pathological or beneficial effects in a certain dose-response relationship (Burtscher et al., 2022). Although it has been hypothesized that the replacement of normoxic periods by hyperoxic periods would lead to the upregulation of specific transcription factors, which may result in a more favourable adaptive response, only few studies have directly compared the effects of intermittent hypoxia-hyperoxia and intermittent hypoxia-normoxia in humans (Serebrovska T et al., 2019; Susta et al., 2020). It has been shown that prolonged exposure (e.g., 24 h) to normobaric hyperoxia (F_i_O_2_ ≥ 0.60) may cause toxicity, which can result in damage to cells and tissues (Mach et al., 2011). Therefore, hyperoxia should also administered with care. To minimize adverse effects, it is recommended to use mild to moderate doses of hyperoxia (Mach et al., 2011; Burtscher et al., 2022). In this regard, Susta et al. (2020) have shown that exposing healthy young males to short-term (one session consisting of 4–6 cycles, lasting 40–50 min) intermittent hypoxia-normoxia (5–7 min of hypoxia [F_i_O_2_ = 0.11] and 3–5 min of normoxia) or IHHE (5–7 min of hypoxia [F_i_O_2_ = 0.11] and 3–5 min of hyperoxia [F_i_O_2_ = 0.30–0.35]) does not increase oxidative stress and does not impair antioxidant defences. This is in line with the protocols applied in the present and previous studies, which used an intensity of hypoxia and hyperoxia ranging from 0.10 to 0.12 F_i_O_2_ and 0.30 to 0.40 F_i_O_2_ lasting 2–7 min and 1–6 min, respectively (Bayer et al., 2017a; Glazachev et al., 2017; Susta et al., 2017; Dudnik et al., 2018; Glazachev et al., 2019; Serebrovska T et al., 2019; Serebrovska Z et al., 2019; Bestavashvili et al., 2021). Moreover, Serebrovska T et al., 2019 reported that 3 weeks of IHHE (4 cycles; 3 min of hypoxia [F_i_O_2_ = 0.12] and 3 min of hyperoxia [F_i_O_2_ = 0.33]) and intermittent exposure to hypoxia-normoxia (4 cycles; 3 min of hypoxia [F_i_O_2_ = 0.12] and 3 min of normoxia) for 5 days a week had positive effects on the metabolic state in patients with prediabetes. Although they have not found advantages of IHHE compared to intermittent hypoxic-normoxic exposure related to these parameters, the authors speculated that one of the advantages of IHHE over intermittent hypoxic-normoxic exposure could be a reduction in the reoxygenation period as normoxic periods were replaced by hyperoxic periods. In this regard, changes in the regulation of pulmonary blood flow in response to hyperoxia have been previously found which may have beneficial effects in terms of pulmonary gas exchange through reduced intrapulmonary arteriovenous shunting (Elliott et al., 2013; Elliott et al., 2014). However, further research is needed to confirm this assumption.

Furthermore, it must be recognized that acute and chronic responses to hypoxia are specific and inter-individual (Manimmanakorn, 2015; Costello et al., 2020). Therefore, the optimal (individual) dose is still unknown. Furthermore, knowing that the individual response (i.e., internal intensity of hypoxia [arterial oxygen saturation or peripheral oxygen saturation]) and not the characteristics of the external environment (i.e., external intensity of hypoxia [oxygen partial pressure or oxygen fraction]) determines an individual’s physiological stimulus, administration of intermittent hypoxia requires an individually tailored response (Törpel et al., 2019; Soo et al., 2020). In consistency with this recognition, the duration and external intensity of each IHHE session was personalized based on the patient’s individual response according to previously published principles and protocols (Glazachev, 2013).

## 5 Limitations

The study has some limitations that have to be considered. The first limitation is the lack of a separate IHHE alone and sham IHHE alone group (i.e., without aerobic cycling exercise). Such groups would allow to uncover whether IHHE alone or in conjunction with aerobic cycling training caused the beneficial effects on patients’ cognitive and physical performance. Second, the training intensity was not controlled, for instance, using a Borg Scale or heart rate as recommended for older adults. There were two reasons for this: 1) in some patients, heart rate was not a suitable index of aerobic intensity due to pacemakers or medications (e.g., beta-blockers) and 2) it was not possible to use a numeric scale because some patients did not comprehend the meaning of this scale. However, an alternative method was chosen, which was previously used in patients with Alzheimer’s dementia to determine the intensity during aerobic cycling training (Holthoff et al., 2015). Third, although both females and males were recruited for this study, only one male was included in the final analysis. Given that sex can influence acute and chronic responses to physical exercise or training and hypoxia (Wadhwa et al., 2008; Barha et al., 2017; Edmunds et al., 2021), the results cannot be generalized to males without limitations. Fourth, patients with and without dementia of different aetiologies were included. However, subgroup analysis has shown that the intervention effects did not change when taking the patients’ level of cognitive impairment into account.

## 6 Conclusion

Hypoxia has already been proposed to be a promising non-pharmacological intervention strategy for improving physical and cognitive performance as well as reducing cardiometabolic risk factors in older adults and geriatric patients (Millet et al., 2016; Bayer et al., 2017a; Glazachev et al., 2019). The current study showed that an additional IHHE prior to aerobic cycling exercise seems to be more effective to increase global cognitive functions as well as physical performance and to preserve functional mobility in geriatric patients in comparison to aerobic exercise alone after a six-week intervention period. Furthermore, the intervention effects did not change when taking the patients’ level of cognitive impairment into account. Therefore, it can be assumed that IHHE is well tolerated and can promote additional effects on cognitive and physical performance in geriatric patients with and without cognitive impairment. The optimal dose and combination of physical exercise or training and IHHE is not yet known. Further investigations are warranted to identify the optimal and individually tailored IHHE regimen and their synergistic effects with physical exercise or training. The underlying (neuro-) physiological mechanisms that cause the improvements in cognitive and physical performance should also be the focus of further research.

## Data Availability

The raw data supporting the conclusion of this article will be made available by the authors, without undue reservation.
