# Supplementary material for: Effects of Intermittent Hypoxia-Hyperoxia Exposure Prior to Aerobic Cycling Exercise on Physical and Cognitive Performance in Geriatric Patients—A Randomized Controlled Trial
Source: Front Physiol. 2022 May 26;13:899096. doi: 10.3389/fphys.2022.899096 (PMC9178199; doi:10.3389/fphys.2022.899096)
Supplement: Supplementary file 1 [file Table1.DOCX]

**Supplementary Table 1:** Within-group differences from baseline in the Dementia Detection Test (DemTect), Clock Drawing Test (CDT), Timed-Up and Go-Test (TUG), and Short-Physical-Performance-Battery (SPPB) and interaction effects according to cognitive status determined though MMSE at baseline.

|  | **Within-group difference** | | |  | **Interaction effect^a^** |
| --- | --- | --- | --- | --- | --- |
| **Measure** | **Exercise** | **n** | **Control** | **n** |  |
|  | **Mean (95% CI)** |  | **Mean (95% CI)** |  |  |
| **DemTect [score]** |  |  |  |  |  |
| Lower (≤ 18) | 1.71 (-0.99 to 4.42) | 6 | 1.00 (-0.15 to 2.15) | 6 | F_1,21_ = 0.003, p = .958,  η_p_^2^ = 0.00 |
| Higher (≥ 19) | 1.33 (-1.12 to 3.79) | 8 | 0.80 (-1.04 to 2.64) | 5 |  |
| **CDT [score]** |  |  |  |  |  |
| Lower (≥ 18) | 1.14 (-0.21 to 2.50) | 6 | 0.17 (-1.23 to 1.56) | 6 | F_1,21_ = 0.076, p = .786,  η_p_^2^ = 0.00 |
| Higher (≤ 19) | 0.67 (-0.60 to 1.94) | 8 | -0.40 (-3.26 to 2.46) | 5 |  |
| **TUG [sec]** |  |  |  |  |  |
| Lower (≥ 18) | -1.49 (-3.12 to 0.15) | 6 | 2.92 (-2.14 to 7.98) | 6 | F_1,20_ = 0.007, p = .936,  η_p_^2^ = 0.000 |
| Higher (≤ 19) | -0.20 (-7.23 to 6.83) | 7 | 4.56 (-4.04 to 13.15) | 5 |  |
| **SPPB [score]** |  |  |  |  |  |
| Lower (≥ 18) | 1.00 (-0.69 to 2.69) | 6 | -0.17 (-0.96 to 0.62) | 6 | F_1,20_ = 0.022, p = .884,  η_p_^2^ = 0.00 |
| Higher (≤ 19) | 1.17 (-0.87 to 3.20) | 7 | 0.20 (-2.02 to 2.42) | 5 |  |

^a^Difference in exercise effect between participants with lower and higher cognitive function at baseline. CI, confidence interval
